# Amperometric determination of nitrogen dioxide in air samples by flow injection and reaction at a gas-liquid interface

**DOI:** 10.1155/S1463924687000105

**Published:** 1987

**Authors:** Frazier W. Nyasulu, Horacio A. Mottola

**Affiliations:** Department of Chemistry Oklahoma State University Stillwater Oklahoma 74078-0447 USA


Journal of Automatic Chemistry/*Journal of Clinical Laboratory Automation*, Vol. 9, No. 1 (January–March 1987), pp. 46–49

### Amperometric determination of nitrogen dioxide in air samples by flow injection and reaction at a gas-liquid interface

**Frazier W. Nyasulu and Horacio A. Mottola***

*Department of Chemistry, Oklahoma State University, Stillwater, Oklahoma 74078-0447, USA*

A number of methods for the determination of $NO_2(g)$ in air by use of spectrophotometric [1 and 2], fluorometric [3], chemiluminescent [4], and electrochemical [5], detection have been reported. These methods require trapping and preconcentration of $NO_2$ as nitrite, a process with unreliable stoichiometry [6], and are either time consuming, or use expensive instrumentation. The method proposed here, while not as sensitive as some of the above, does not require expensive instrumentation nor pretrapping steps and thus allows for determination of real-time $NO_2$ concentrations.

The determination is based on the $NO_2$ oxidation of tris(1,10-phenanthroline)-iron(II) [ferroin] to its iron(III) analogue at a gas-liquid interface. Two continuous-flow manifolds for sample processing are described and compared. One incorporates a gas diffusion step in which a gas-permeable membrane separates a donor (sampling) stream from an acceptor (detecting) stream. Some of the air sample containing $NO_2$ diffuses across the membrane into the detecting stream and there causes ferroin oxidation. In the other manifold, the $NO_2$-air sample is directly intercalated into the ferroin-containing liquid carrier; oxidation occurs at the gas-liquid interface. The extent of ferroin oxidation is amperometrically monitored at a carbon paste electrode and is proportional to the $NO_2$ concentration in the air sample. The ferroin/ferriin redox couple is a reversible electrochemical system in which oxidation/reduction occurs without affecting the chemical structure of the 1,10-phenanthroline ligand. Some information on the electrochemistry of the ferroin/ferriin couple can be found in the literature [7]. An advantage of the reversibility of this redox process is that the ferriin produced during the oxidation with $NO_2$ can be electrochemically reduced back to ferroin in a reagent reservoir and thereby can be used in a closed-loop system [8]. A similar direct intercalation procedure for the determination of $SO_2(g)$ has been reported [8,9] in which $SO_2(g)$ reduces di-μ-hydroxo-bis[bis(1,10-phenanthroline)iron(III)] at a gas-liquid interface. The product of the reduction, ferroin, can be photometrically monitored at 510 nm [8] or amperometically at 965 mV (versus an Ag/AgCl reference electrode) [9].

*Author to whom correspondence and reprint requests should be addressed.

### Experimental

### *Apparatus*

A schematic of the gas diffusion flow-injection system is shown in figure 1(*a*). The flowing streams were propelled by a Minipuls 2 peristaltic pump (Gilson Medical Electronics, Middleton, Wisconsin, USA). Sample intercalation was accomplished by use of a Rheodyne rotary four-way valve (Type 50). A representation of the custom made gas diffusion cell is shown in figure 1(*b*). The gas-permeable membrane used, of 0·45-μm nominal pore size, was from Garlock Inc. (Newton, Pennsylvania, USA). Figure 1(*c*) shows a representation of the amperometric detection cell. The carbon paste working electrode and the platinum auxiliary electrode were placed so that they were 1 mm apart, this being the diameter of the Teflon connecting tubing used. The reference electrode was an SCE. The carbon paste was prepared by thorough

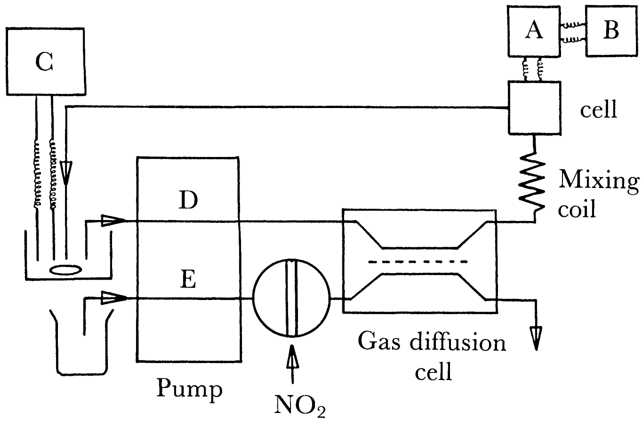

*Figure 1(a). Configuration of the gas diffusion flow injection analysis system A: Potentiostat, B: Strip chart recorder, C: Potentiostat for reagent regeneration, D: Ferroin solution in 1·0 M KCl, E: 1·0 M KCl.*

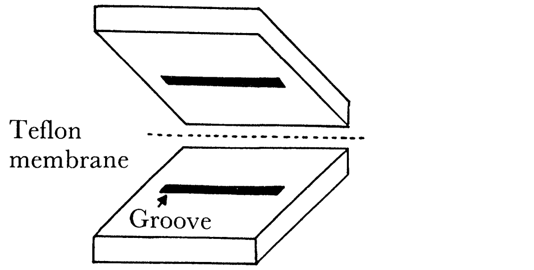

*Figure 1(b). Gas diffusion cell. Groove dimensions in each block were 6 cm long, 2 mm wide and 0·2 mm deep.*

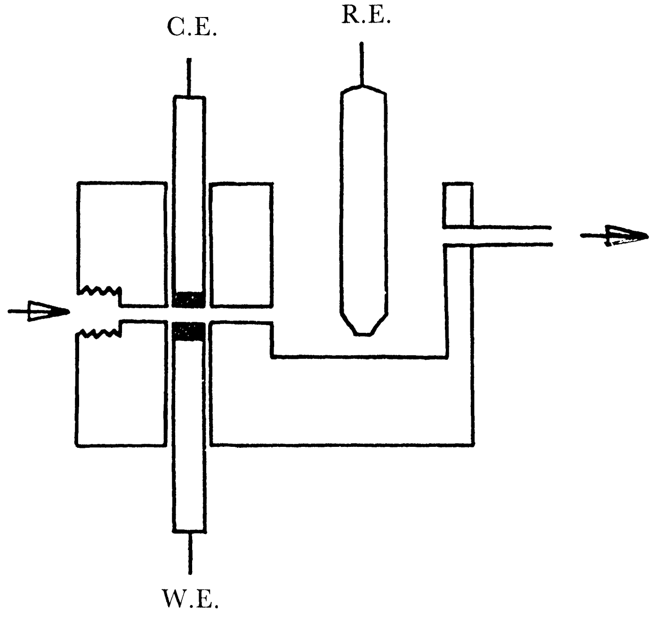

*Figure 1(c). Amperometric flow cell.*

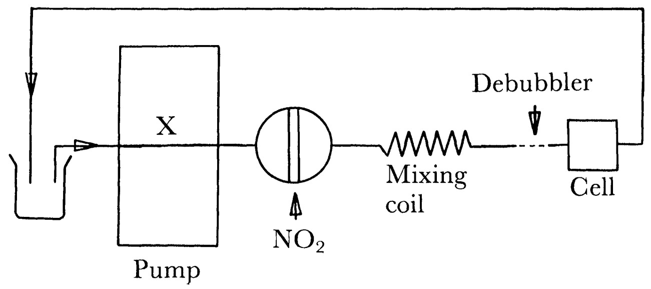

*Figure 2. Configuration for the direct intercalation procedure, X = ferroin in 1·0 M KCl.*

mixing of 1·0 g of UCP-1-M graphite from Ultra Carbon (Bay City, Michigan, USA), 0·5 g of hexadecane (Aldrich Chemical Co., Milwaukee, Wisconsin, USA), and 0·5 g of high-vacuum grease (Dow Corning Corp., Midland, Michigan, USA). The platinum electrode was from Bioanalytical Systems (West Lafayette, Indiana, USA). A custom-built potentiostat [10] was used for amperometric determination with its output monitored by a Hewlett-Packard Model 7128A strip-chart recorder. A MPI 1026 potentiostat-regulated power supply was used to convert the oxidation product, ferriin, back to the main reagent, ferroin, by electrolysis at 650 mV *vs* SCE.

The direct intercalation system is shown in figure 2. A 5 cm length of Gore-Tex microporous PTFE tubing (1 mm i.d.) of 3·5 μm pore size (Anspec, Ann Arbor, Michigan, USA) was used as a debubbler [11]. This tubing functioned best in the flow system when placed vertically with the solution flowing downwards. The tube between the gas diffusion cell and detector in figure 1(*a*) and injector to detector in figure 2 had a single-bead-string-reactor configuration produced by incorporation of glass beads 0·6 mm in diameter inside 1 mm i.d. tubing. Such reactors enhance the mixing performance in flow injection systems [8 and 12].

*Reagents and solutions*

All reagents used were of AR grade. Deionized-distilled water was used for solution preparations. Tris(1,10-phenanthroline)iron(II), in the form of a 0·025 M aqueous solution was obtained from G. F. Smith Chemical Co. (Columbus, Ohio, USA). This ferroin solution was diluted with 1·00 M aqueous KCl to obtain the desired concentrations. The $NO_2(g)$ samples were prepared daily prior to use by dilution of compressed $NO_2(g)$ (Matheson Gas Products, La Porte, Texas, USA) with air.

*Procedure*

Prior to use for detection, the carbon paste electrode was immersed in a 0·10% (w/v) stirred solution of Triton-X-100 (Rohm & Haas, Philadelphia, Pennsylvania, USA) for 10 min. This treatment has been shown to improve the electrochemical response of carbon paste electrodes [13]. Before intercalation of the $NO_2$-air sample the detector was allowed to reach equilibrium. A gas-tight syringe (Precision Sampling Corp., Baton Rouge, Louisiana, USA) was used to introduce the sample into the sample loop of the rotary valve. In the direct intercalation, it was necessary to flush out the ferroin solution, in the sample loop with 1·0 M KCl prior to sample introduction. Without this procedure, $NO_2$ reacts with the ferroin film coating the inside of the sample loop and the signals obtained depend not only on $NO_2$ content of the sample but also on its residence time in the sample loop and amount of gas passed to waste.

### Results and discussion

*Determinations by gas diffusion*

The effect of varying the detection potential from 700 to 900 mV *vs* SCE is shown in table 1. These measurements were made with an $NO_2$ concentration (v/v) of 100 ppm, a 70-μl sample volume, a 1 ml/min flow rate, a 25 cm length of reactor tubing from the gas diffusion cell to the detector, and a 1·00 × $10^{-5}$ M ferroin solution (pH = 5·00). From the table it is clear that the optimum detection potential, with respect to signal size, is between 780 and 900 mV. It was found, however, that a substantial increase in base-line noise resulted when potentials in excess of 800 mV were used. Consequently all subsequent measurements were made at an applied potential of 780 mV. At this potential, the base-line current corresponds to the oxidation of ferroin to ferriin.

*Table 1. Effect of detection potential on signal height with a 100 ppm $NO_2$ sample. See text for experimental conditions.*

| Potential (mV) | Current (nA) |
|---|---|
| 700 | 0 |
| 730 | 3 |
| 750 | 7 |
| 780 | 12 |
| 800 | 13 |
| 850 | 14 |
| 900 | 13 |

The signals recorded reflect the decrease in the current as a result of ferroin oxidation by the $NO_2$ contained in the intercalated sample.

Determination of $NO_2(g)$ based on the detection of the oxidation product (ferriin) is possible at potentials less than 630 mV, however, the sensitivity and precision of such measurements was poor when compared to measurements at 780 mV (ferroin detection).

Figure 3 shows the effect of varying the length of reactor tubing from the gas diffusion cell to the detector. The optimum length, at a flow rate of 1 ml/min, fell in the range of 20–25 cm. Shorter lengths resulted in insufficient time for signal realization and greater lengths produced substantial dispersion (dilution).

Table 2 shows the effect of varying the intercalated sample volume from 70 to 350 μl. A volume of 245 μl was chosen for all further measurements. Larger volumes decreased the sample frequency without appreciable increase in sensitivity.

A systematic study of the effects of ferroin concentration and hydrogen ion concentration led to the choice of $1{\cdot}00 \times 10^{-5}$ M ferroin and pH = 5·00.

Figure 4 shows typical signal profiles using the conditions chosen as best. The equation for calibration curves derived from these signals was:

$$i \text{ (current in nA)} = 0{\cdot}3\,[NO_2(g)] - 0{\cdot}2$$

with the $NO_2(g)$ concentration in ppm (v/v) and a correlation coefficient of 0·9994. The limit of detection (defined as the average of blank signals plus three times its standard deviation) was found to be 12 ppm. Ten successive measurements of 50 ppm $NO_2$ gave a relative standard deviation of 2·1%. Determination could be made at a sampling frequency of 35/h. The use of air carrier instead of a liquid one resulted in smaller signals, presumably because of a larger dispersion (dilution) taking place between the point of intercalation and the gas diffusion cell.

*Determinations by direct intercalation*

Ferroin concentration, pH, detection potential, flow rate, and sample volume were the same as those used in the gas diffusion determinations. A 1.0 m length of tubing was used from intercalator to detector.

*Table 2. Effect of sample volume on signal height; $NO_2$ concentration = 100 ppm, ferroin concentration = $1{\cdot}00 \times 10^{-5}$ M (pH = 5), flow rate = 1 ml/min, gas diffusion cell to detector distance = 20 cm.*

| Volume (μl) | Current (nA) |
|---|---|
| 70 | 12 |
| 90 | 20 |
| 170 | 25 |
| 210 | 26 |
| 245 | 28 |
| 300 | 29 |
| 350 | 29 |

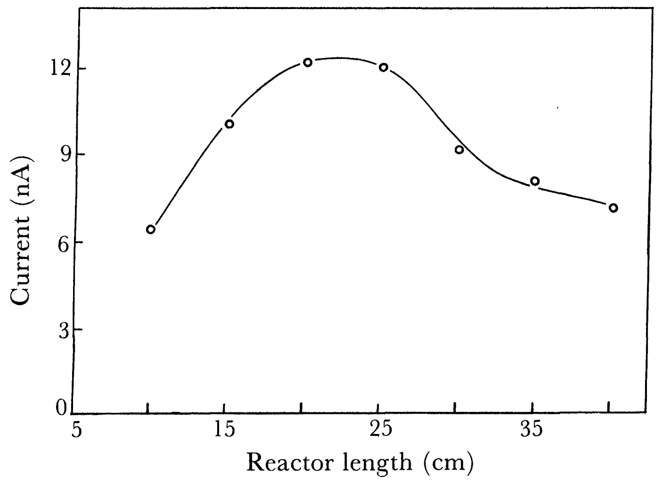

*Figure 3. Effect of reactor length on signals obtained with 100 ppm $NO_2$.*

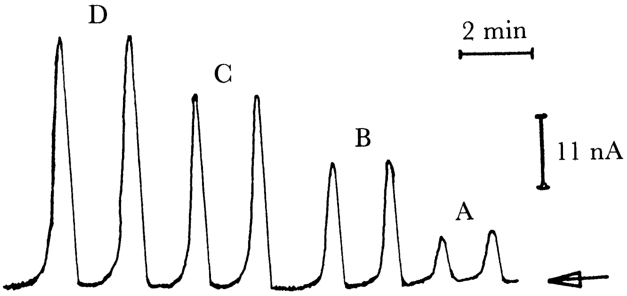

*Figure 4. Gas diffusion flow injection profiles, A = 30 ppm, B = 85 ppm, C = 125 ppm, D = 140 ppm.*

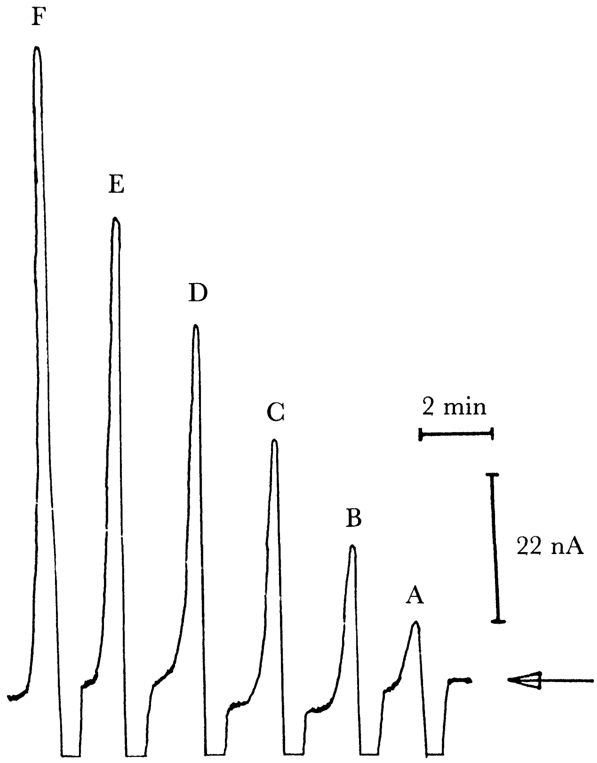

*Figure 5. Direct intercalation flow injection profiles, A = 1·0 ppm, B = 2·8 ppm, C = 4·6 ppm, D = 5·8 ppm, E = 7·6 ppm, F = 10·6 ppm.*

Figure 5 shows typical flow injection profiles recorded in the range of 1·0 to 10·6 ppm by the direct intercalation procedure. The negative peaks partially cut by positioning of the recorder pen occurred because the gas sample reached the debubbler and disturbed the flow continuity. No flow throughout the detector was sustained during the exit of gas through the debubbler. Amperometric detection is known to be sensitive to flow fluctuations [14]. The following equation describes the calibration curve derived from signals obtained by direct intercalation:

$$i \text{ (Current in nA)} = 10{\cdot}2\ [NO_2(g)] - 0{\cdot}3$$

with a correlation coefficient of 0·9998. The limit of detection was found to be 0·5 ppm. Ten successive determinations of 4·5 ppm $NO_2$ (v/v) gave relative standard deviation of 2·9%. Determinations could be made at a frequency of 25 samples/h.

This intercalation procedure is more sensitive than the gas diffusion one for three reasons: (1) there is a longer contact time between the air sample and the ferroin; (2) the contact area for the $NO_2$-ferroin reaction is larger; and (3) the gas diffusion membrane, which introduces a rate limiting effect, is absent.

Hydrogen sulphide present in concentrations of about 300 ppm (v/v) caused no interference. Larger concentrations of this gas introduced a positive interference. This interferent effect is due to $H_2S$ reduction at the working potential [15] and can be eliminated by making measurements in presence and absence of ferroin. Chlorine, on the other hand, causes serious negative interference for unknown reasons.

### Acknowledgement

Research support from the Office of Basic Energy Sciences (Grant DE-FG05-85ER 13346) of the US Department of Energy is gratefully acknowledged here.

### References

1. Hauser, T. R. and Shy, C. M., *Environmental Science and Technology*, **3** (1972), 890.

2. Saltzman, B. E., *Analytical Chemistry*, **26** (1954), 1949.

3. Axelrod, H. D., Pickett, R. A. and Engel, N. A., *Analytical Chemistry*, **47** (1975), 2021.

4. Hodgeson, J. A., McClenny, W. A. and Hanst, P. L., *Science*, **182** (1973), 248.

5. Suzuki, S. D. and Natashima, K., *Analytica Chimica Acta*, **144** (1982), 261.

6. Harrison, R. M., *CRC Critical Reviews in Analytical Chemistry*, **15** (1985), 1.

7. Ogura, K. and Miyamoto, K., *Electrochimica Acta*, **22** (1977), 1357.

8. Ramasamy, S. M. and Mottola, H. A., *Analytical Chemistry*, **54** (1982), 283.

9. Rios, A., Luque de Castro, M. D., Valcarcel, M. and Mottola, H. A., *Analytical Chemistry*, in press.

10. Albahadily, F. N. and Mottola, H. A., *Journal of Chemical Education*, **63** (1986), 271.

11. Martin, G. B., Cho, H. K. and Meyerhoff, M. E., *Analytical Chemistry*, **56** (1984), 26129.

12. Reijn, J. M., Van der Linden, W. E. and Poppe, E., *Analytica Chimica Acta*, **123** (1982), 229.

13. Albahadily, F. N. and Mottola, H. A., *to be published.*

14. Wolff, Ch.-M. and Mottola, H. A., *Analytical Chemistry*, **50**, (1978), 94.

15. Nygaard, D. D., *Analytica Chimica Acta*, **127** (1981), 257.

### THE CHEMICAL SENSORS CLUB

The Chemical Sensors Club has been formed to ensure that all organizations in the UK concerned with the exploitation of chemical sensor technology are as well co-ordinated as possible.

The advantages of Club membership are:

(1) The Club provides a forum through which member organizations can make known their capabilities and needs.
(2) Two general meetings are organized each year to enable members to discuss particular aspects of chemical sensor technology and to establish personal contacts with each other.
(3) A members' handbook contains printed information generated or compiled by the Club covering current areas of interest of member organizations, available hardware, UK research activities, details of consultancy services and any other more detailed reports on specific aspects of sensors.
(4) Members also receive a regular newsletter covering Club activities and topical items of interest in the sensors field.
(5) Data-bases are being established to allow members to gain access to collated information on user needs, available sensor systems and research activities in progress.
(6) Club membership may entitle members to reduced subscription rates for other DTI funded groups active in the sensors field.
(7) Through membership of the Club, organizations will be assisted to join together to form consortia, specific to their interests, to develop new chemical sensors and sensor applications.

*Details of membership from Mr D. G. Porter, Laboratory of the Government Chemist, Cornwall House, Waterloo Road, London SE1 8XY.*

